# A tightly regulated and adjustable CRISPR-dCas9 based AND gate in yeast

**DOI:** 10.1093/nar/gky1191

**Published:** 2018-11-22

**Authors:** Anja Hofmann, Johannes Falk, Tim Prangemeier, Dominic Happel, Adrian Köber, Andreas Christmann, Heinz Koeppl, Harald Kolmar

**Affiliations:** 1 Institute for Organic Chemistry and Biochemistry, Technische Universität Darmstadt, 64287 Darmstadt, Germany; 2 Institute of Condensed Matter Physics, Technische Universität Darmstadt, 64289 Darmstadt, Germany; 3 Department of Electrical Engineering and Information Technology, Technische Universität Darmstadt, 64283 Darmstadt, Germany

## Abstract

The robust and precise on and off switching of one or more genes of interest, followed by expression or repression is essential for many biological circuits as well as for industrial applications. However, many regulated systems published to date influence the viability of the host cell, show high basal expression or enable only the overexpression of the target gene without the possibility of fine regulation. Herein, we describe an AND gate designed to overcome these limitations by combining the advantages of three well established systems, namely the scaffold RNA CRISPR/dCas9 platform that is controlled by Gal10 as a natural and by LexA-ER-AD as heterologous transcription factor. We hence developed a predictable and modular, versatile expression control system. The selection of a reporter gene set up combining a gene of interest (GOI) with a fluorophore by the ribosomal skipping T2A sequence allows to adapt the system to any gene of interest without losing reporter function. In order to obtain a better understanding of the underlying principles and the functioning of our system, we backed our experimental findings with the development of a mathematical model and single-cell analysis.

## INTRODUCTION

Synthetic biology aims at designing modular genetic circuits that combine and interconnect different logic gates. A key requirement for gate construction is a reliable and well-defined switching characteristic in living organisms as well as minimal crosstalk between different gates. Regulation systems based on natural transcription machineries such as the galactose activated Gal80/Gal4 system have been used for decades to control the gene expression in *Saccharomyces cerevisiae* (1). Nevertheless, natural transcription factors influence cell growth and depend on host cell genes for correct performance (2–4). Another approach relies on the use of synthetic transcription factors such as e.g. zinc fingers. Though these transcription factors do not considerably influence cell growth, each one has to be designed individually to target a specific locus (5). Hence, recent research efforts rely on the design of individually inducible genetic switches that show low basal activity, high levels of transcription activation and preferentially no toxicity (6,7).

**Figure 1.**
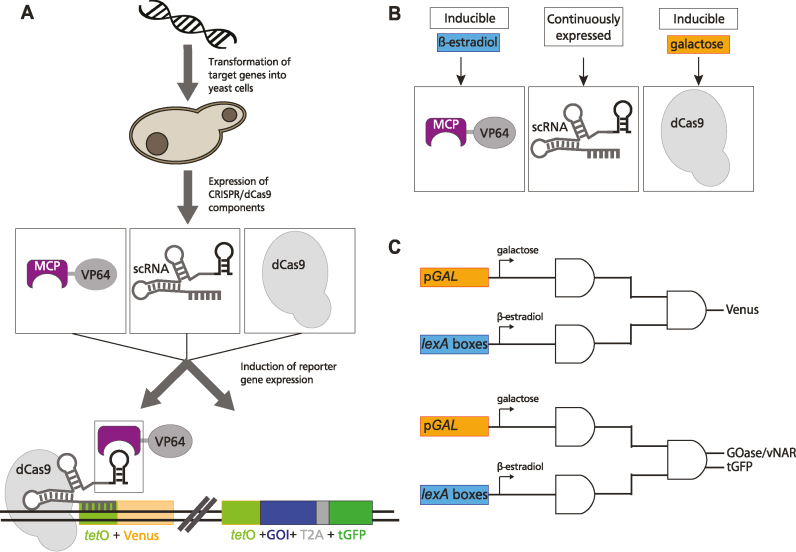
(**A**) Schematic outline of a genetic switch based on dCas9-mediated transcription activation. We used two different reporter systems: (A, lower left) Single reporter system using a yellow fluorophore (Venus) as reporter. Expression requires inducers galactose and β-estradiol. (A - lower right) Double reporter system containing a combination of GOase (any other gene of interest (GOI) is possible) and tGFP as reporters connected by a T2A peptide. (**B**) Regulatory components for reporter gene expression. Expression of two of the three parts, namely MCP-VP64 and dCas9, is dependent of added inducers β-estradiol and galactose. (**C**) The AND gate consists of two independent switches, which both must be activated to enable reporter gene expression.

Recently, usage of the bacterial CRISPR system in an eukaryotic host was shown to overcome the aforementioned limitations and to provide a useful platform for the design of distinct controllable genetic switches (8–10). To this end, a single-guide RNA (sgRNA) is used to target a catalytically inactive Cas9 (dCas9) to specific loci. By fusing dCas9 to transcription activating modules, domain specific switches can be installed. Zalatan *et al.* extended the conventionally used sgRNA to a scaffold RNA (scRNA) that provides target specificity as well as regulatory function. The scRNA possesses not only the hairpin loop for dCas9 recruitment but also a modular RNA domain. This RNA domain recruits RNA-binding proteins like the bacteriophage coat protein MS2 fused to a transcription regulator as e.g. KRAB or VP64 (Figure 1A). As a consequence, simultaneous multi-directional regulation of different target genes is possible, which is the basis for any modular switch system (8–11).

Making the expression of dCas9 controllable by an inducible *pGal10-dCas9* construct, Zalatan *et al.* were able to use the scRNA CRISPR/dCas9 system to build a galactose inducible switch. While showing high activation levels and good specificity, the system has due to the induction by galactose the shortcoming of not being controllable in a fast and fine-tuned fashion (4,10).

In order to establish a generic modular platform based on the scRNA CRISPR/dCas9 system that is also precisely tunable with low induction levels, as well as tightly regulated, we placed the expression of scRNA-binding fusion protein MCP-VP64 under tight control of β-estradiol (ES) using a promoter that contains several *lexA* boxes and a heterologous transcription factor comprising the ES-binding domain of the human estrogen receptor (ER) (12–14), the bacterial LexA DNA-binding protein and the activation domain (AD) B112 aimed at allowing precise adjusting with β-estradiol as inducer (Supplementary Figure S1) (2).

Hence, by combining the sensitive LexA based β-estradiol tunability with the modularity and low basal activity of CRISPR/dCas9 we designed an AND gate depending on both galactose and ES. Additionally, due to the low induction concentrations, we minimized possible but unwanted side effects. We aimed to establish a tightly regulated, sensitive and modular system, which allows the easy exchange of the gene of interest (GOI). Due to a minimal mathematical model, that—despite its simplicity—is able to cover all the relevant effects observed, we could predict and explain the observed switching behavior.

Having in mind possible biotechnological applications, we investigated switching behavior upon the controlled expression of the enzyme galactose oxidase (GOase) using a T2A-tGFP. Switching behavior was investigated by bulk and single cell analysis. This type of AND gate may enable the development of more complex, but still reliable logic gates.

## MATERIALS AND METHODS

### Yeast strains, media and plasmids

The yeast strain cSLQ.Sc002 used in this study was derived from *S. cerevisiae* (10). Cells were grown at 30°C in complete medium with 2% glucose (YPD) or synthetic complete medium with 2% glucose (SD), respectively. Strain constructs are listed in Supplementary Table S1 and plasmids in Supplementary Table S2.

### DNA preparation

For switch construction, DNA was genomically integrated using the CasEMBLR method (15). *Escherichia coli* strain DH5α was used for plasmid preparation. Polymerase chain reaction (PCR) was performed following the manufacturers instructions using either Q5^®^High-Fidelity DNA Polymerase or Phusion^®^ High-Fidelity DNA Polymerase (New England Biolabs, Inc.). To allow assembly and integration via homologous recombination *in vivo* 30 bp overlaps were added to the ends of each part when combined with another part and 120 bp when combined with the yeast genome. All oligos are listed in Supplementary Tables S3 and S4. The DNA part size was confirmed by agarose gel electrophoresis, followed by purification using Wizard^®^ SV Gel and PCR Clean-Up System (Promega GmbH). To perform *in vivo* one-step assembly and genomic integration via homologous recombination 4 pM of each part were mixed, unless parts were longer than 6000 bp and 1 μg DNA was used instead. For single locus integration 2 μg of corresponding gRNA plasmid or for integration in multiple loci 1 μg of each gRNA plasmid was added. The mixture was concentrated by ethanol precipitation and resuspended in 5–10 μl of Milli-Q water.

### Yeast strain construction

Cells of the strain cSLQ.Sc002 were made chemo-competent using the Frozen-EZ Yeast Transformation II Kit (Zymo Research) and transformed with 2 μg of the Cas9 encoding plasmid p414 (15). Transformants were selected by SD-TRP agar plates and integration was checked by colony PCR. Correct cSLQ.Sc002_p414 clones were picked, made chemo-competent again and used for following chromosomal integrations. Transformation with complete mixture of desired parts and corresponding gRNA(s) was performed using these chemo-competent cSLQ.Sc002_p414 cells. After transformation, cells were plated on SD-TRP-LEU agar plates and clones were checked by colony PCR for correct part assembly, as well as for correct genomic integration. The CasEMBLR plasmids were removed by growth in regular YPD and subsequent dilution of the culture to 5 × 10^5^ cells/ml. One hundred microliters of cells were plated out on YPD agar plates and grown for 2 days. Single colonies were picked and transferred to YPD, SD-TRP and SD-LEU agar plates and grown again. Clones only growing on YPD were selected for further experiments. The plasmid free clones were transformed with 2 μg of scRNA encoding plasmid (*URA3* marker) according to a protocol of Benatuil *et al.* (16). Cells were plated out on SD-URA agar plates and correct plasmid uptake was checked by colony PCR and function.

### Cytometry

The fluorescence intensity of Venus and tGFP was measured with a BD Accuri C6 flow cytometer using excitation wavelengths of 488 nm and an emission detection filter at 533 nm (FL1 channel). On average 50 000 cells were recorded for each sample. For analysis, cytometry data were exported as FCS 3.0 files and processed using Mathematica 11 software. In order to maintain comparability and comprehensibility, we followed a minimal gating strategy and used only two constant gates to exclude debris as well as possible doublets. The exact gating is described in Supplementary Section SI3.1 and Supplementary Figure S13.

### Data collection for induction control and stepwise induction

Yeast cells were grown in SD-URA medium overnight at 30°C. The cell density was determined photometrically and 1 × 10^7^ cells/ml were inoculated in synthetic complete medium with 2 % galactose (SG) leaking uracil. Induction was completed by addition of ES. For a stepwise induction the overnight culture was aliquoted into prepared SG-URA medium, ES was added at different concentrations and cells were grown in glass tubes with high oxygen contact at 25°C (17,18). Samples were analyzed after 20 h of induction. Therefore, 300 μl of the cell culture were centrifuged at 8000 rpm in a Heraeus Biofuge Pico centrifuge to remove medium. Cells were resuspended in phosphate buffered saline (PBS) and analyzed by flow cytometry followed as described above. Data analysis was performed using Mathematica 11 software.

### Data collection for time dependent measurement

For time dependent measurement, 10 ml of SG-URA medium were inoculated with yeast cells as described above. For induction either 10 or 100 μl of a 10 μM ES stock solution were added for induction with a concentration of 10 or 100 nM, respectively. Cells were grown at 25°C for 48 h. After each hour 200 μl of cells were removed and 200 μl of SG-URA medium containing 10 nM/100 nM ES were added. The cell suspension was centrifuged, the supernatant was used for ABTS assay (see ‘Materials and Methods’ section - Data collection for activity of GOase) and the cells were suspended in PBS for flow cytometry measurement. Data analysis was performed using Mathematica 11 software.

### Preparation of GOase

Cells were grown overnight in 50 ml flasks in SD-URA medium at 30°C. After growth cells were inoculated to a cell density of 1 × 10^7^ cells/ml into 1000 ml SG-URA and induction was completed by addition of 1000 μl of a 1 mM ES stock solution. Induction was performed at 25°C for 20 h. Induction of GOase-tGFP expression was checked by flow cytometry. The cells were precipitated and the supernatant was concentrated to 25 ml using a Vivaflow 200, 10.000 MWCO Hydrosart (Sartorius) laboratory crossflow cassette. The cell pellet was resuspended in PBS, disrupted using a cell disrupter (Constant systems LTD) and cell debris was removed by centrifugation. Both supernatant and cell debris were analyzed using sodium dodecyl sulphate-polyacrylamide gel electrophoresis (SDS-PAGE) as well as ABTS assay. SDS-PAGE was performed as described in (19).

### Activity measurement of GOase

ABTS (2,2′-azino-bis(3-ethylbenzothiazoline-6-sulphonic acid)) assay was used to determine GOase activity. To this end, 180 μl of cell suspension or supernatant (sample diluted 1:10 in PBS) were mixed with 10 μl Horseradish peroxidase (0.1 mg/ml), 10 μl ABTS (10 mM) and 10 μl galactose substrate (1 M) (20,21). After 5 min the absorption of ABTS was measured at 405 nm with INFINITE Tecan M-1000 (Tecan). Absorption was exported to MS Excel (Microsoft) and analysis was performed using Mathematica 11 software.

### Single-cell microfluidics and time-lapse microscopy

Time-lapse single-cell experiments were carried out on a microfluidic platform inspired by ALCATRAS (22). Cells containing the GOase-2A-tGFP expression cassette were precultured at 30°C for 5 h in SD-URA media. At *t* = 0 h the cells were induced with both SG-URA media and ES concentrations of 20 or 100 nM, respectively. Once loaded onto the microfluidic chip, the immobilized cells are exposed to a controlled environment in continuous flow of the respective inducer medium ($\dot{V} = 10\, \mu \textrm{l}/\textrm{min}$). The flow ensures that daughter cells are flushed away, while the cells of interest are confined to the traps. Microscope images were recorded every 10 min at five different focal planes (temperature: 30°C; microscope: Nikon Eclipse TI; objective: 60×, camera: Hamamatsu ORCA Flash4.0; light source: Lumencor SpectraX). Single traces were extracted with a FiJi/Matlab script, where only the original cells were measured and daughter cells were omitted. Traces are corrected for each frame’s background fluorescence and each cell’s individual background fluorescence at *t* = 4 h. A moving average filter with a window size of 3 was employed to reduce noise for the purpose of visualization.

### Mathematical modeling

We developed a minimal model that captures the most processes and is still capable of covering all important results of the experiment. The model consists of three reactions, namely the diffusion process of the β-estradiol–transcription factor complex (ES), the ES activated expression and the degradation/dilution of the GFP messenger RNA (mRNA) (G):

(1) \begin{eqnarray*} {\rm Diffusion:} \; \rm{ES_m} \mathop{\rightleftharpoons}^{\gamma } \rm{ES_n} \nonumber \\ {\rm Expression:} \; \emptyset \mathop{\longrightarrow}^{f(\rm{es_n})} \rm{G} \\ {\rm Degradation/Dilution:} \; \rm{G} \mathop{\rightarrow}^ {\delta } \emptyset \nonumber \\ {\rm where: } f({\rm es_n}) = \frac{\nu \; \left({\rm es_n}/k\right)^h}{1 + ({\rm es_n}/k)^h}\nonumber .\end{eqnarray*}

Here, ES_n_ and ES_m_ denote the TF-Complex in and outside of the nucleus, respectively. The used Hill-Function *f*(es_n_) comprises the Michaelis constant *k*, the Hill-Coefficient *h*, as well as the maximum expression factor *ν* and depends on the concentration of ES in the nucleus. The parameters *δ* represents the degradation/diffusion of the mRNA and *γ* captures the diffusion rate of ES.

Based on this reaction equations, we generated a system of two ordinary differential equations that describe the time dependent concentrations of the β-estradiol–transcription factor complex in the nucleus(es_n_) and the GFP mRNA (g):

(2) \begin{equation*} \frac{{\mathrm{d}} \mathrm{es_n}(t)}{{\mathrm{d}} t} = -\mathrm{es_n}(t) \, \gamma + \mathrm{es_m}(t) \, \gamma \end{equation*}

(3) \begin{equation*} \frac{{\mathrm{d}} \mathrm{g}(t)}{{\mathrm{d}} t} = -\mathrm{g}(t) \, \delta + f(\mathrm{es_n}(t)). \end{equation*}

The concentration of ES in the medium (es_m_) is given by the experimental conditions and is held constant during the experiment. To be able to compare the model and the data, we introduced a scaling factor Φ, that accounts for the translation process and additionally maps the number of fluorescent proteins to the measured fluorescence value.

The obtained values are given in the Supplementary Tables S5 and S6.

The deterministic model was fitted to the stationary dose–response data to obtain values for *h* and *k*. This fitting was conducted using a pseudo-Newton Algorithm. Since the two reporter systems are based on different fluorescent proteins, we performed separate fits for each of them. Based on the reaction Equations (1) and adopting the parameters obtained by fitting of the deterministic model, we build a stochastic model (detailed data handling described in Supplementary Section SI3.5). We simulated the system using the Stochastic Simulation Algorithm (23) of the Dizzy Software Package (24).

In order to account for the detrimental effects observed in the dual-reporter system, we introduced a generic function *ζ*(ES). The variable *ζ* depends on the ES-concentration and predicts a fraction of cells that do not show any fluorescence. According to this fraction, we set randomly chosen data-points to zero and rescaled the remaining distribution (detailed explanation in Supplementary Section SI3.6)

## RESULTS

### Design of scRNA CRISPR/dCas9 platform with AND gate implementation

CRISPR proved to be a toolbox for a wide variety of applications and therefore many systems for active and inactive Cas9 were designed (25–27). A minimal sgRNA consists of a variable 20 nt DNA targeting sequence and two structured RNA domains for dCas9 recruitment and 3’tracrRNA for proper structure formation (28–30). Instead of a sgRNA, we used a scRNA, which was extended by an additional loop containing the viral sequence MS2 that binds specifically to a *tet*O promoter region (31) (Precise scRNA construction in Supplementary Figure S2). The two other parts were the RNA-binding protein MCP fused to the transcriptional activation domain VP64 (32) as well as a dCas9. This arrangement was shown by Zalatan *et al.* to allow expression and repression of reporter genes in versatile ways in *S. cerevisiae* as well as in mammalian cells and offers the possibility to work with different components on DNA and RNA level (10).

All genes except the scRNA, which was integrated in *S. cerevisiae* as a CEN/ARS plasmid, were integrated into the yeast genome together with *tet*O promoter driven reporter genes differing in the number of the *tet*O boxes (Figure 1A).

We designed a logic AND gate depending on two independently switchable parts, namely dCas9 and MCP-VP64 expression that were controlled by galactose or β-estradiol addition, respectively (Figure 1B). To this end, dCas9 was expressed under control of the galactose-inducible *GAL10* promoter as described (4,10). The inducible expression of dCas9 showed robust reporter gene expression depending on the *tet*O copy number (Supplementary Figure S3), but high basal activity was observed (Supplementary Figure S4).

To reduce the basal activity of the galactose only switch and to enable precise fine tuning, we added a second switchable part controlled by β-estradiol (ES) as published by Ottoz *et al.* (2). To this end, four *lexA* boxes were placed in front of the MCP-VP64 fusion protein coding sequence and a constitutively expressed gene for the heterologous transcription factor LexA-ER-B112 was added, which was shown to have no toxic effects on yeast cells (Supplementary Figure S5). As a consequence, an AND gate should be implemented that is active only in the presence of both ES and galactose combining the robust transcription activation of the *GAL10* system and the sensitive tunability of the ES system (Figure 1C).

All parts except the plasmid-encoded scRNA were integrated into the yeast genome. Integration was performed using the CasEMBLR method (15). The MCP-VP64 under control of four *lexA* boxes and the dCas9 coding sequence under control of the *Gal10* promoter were integrated into the locus *ADE2* (Supplementary Figure S6A). The reporter gene under control of a 7x *tet*O promoter region and the LexA-ER-B112 fusion protein were integrated into the *HIS3* locus (Supplementary Figure S6B and C). In a second transformation step, the scRNA plasmid was introduced via a CEN/ARS plasmid. This approach allowed the spatial separation of transcription factor and switch, as well as the fast and easy exchange of different parts like promoters or terminators or even of the GOI.

### Reporter gene design and construction

Two reporters were used to allow precise characterization of the AND gate. As a single reporter, the yellow fluorescent protein Venus was implemented behind the *tet*O promoter region (Figure 1C). All parts were integrated as described above.

As a more complex setup, a dual reporter system was implemented for which two reporter genes were connected by the picornaviral 2A peptide. The 2A peptide allows for the generation of multiple proteins from one mRNA transcript by ribosomal skipping (33,34). We chose to use the 18 amino acid T2A sequence derived from *Thosea asigna* virus. In comparison with other 2A variant, the T2A sequence results in the most efficient cleavage (35). After Grzeschik *et al.* the usage of T2A allows one to monitor expression of secreted or cell surface displayed proteins by measurement of fluorescence of a 2A coupled GFP that remains in the cell cytoplasm (36).

To investigate, whether expression monitoring works with both inter- and extracellular protein expression, we constructed a GOase-tGFP double reporter system. The T2A sequence was used to combine a secreted GOase from *Fusarium spec*. with the green fluorophore turbo GFP, which should remain in the cell after ribosomal skipping. In addition to indirectly monitoring enzyme production by measuring cellular green fluorescence, the GOase mediated oxidative conversion of D-galactose to D-galacto-hexodialdose can easily be followed by quantification of hydrogen peroxide formation (37,38). To investigate, whether translational readthrough occurs, which would result in the synthesis of a fusion protein rather than the synthesis of two translation products, another construct was made, where the GOase coding sequence is replaced by vNAR-6His (Supplementary Figure S6D, a shark derived antibody domain with C-terminal hexahistidine tag that can be used for protein detection by western blot analysis (39). Reporter genes were genetically implemented after 7x *tet*O via CasEMBLR (Figure 1A).

### AND gate is functional and tightly regulated

We used both reporter constructs in parallel to characterize the behavior of the AND gate. First, the general functionality of the gate was demonstrated. As described by Ottoz *et al.* the LexA-ER-B112 mediated gene expression reaches steady state from 0 to 2000 nM ES concentration (2). For both newly designed reporter systems our model predicts maximum reporter gene expression at a concentration above ∼35 nM of ES after 20 h of induction. Hence, we chose 100 nM ES as maximum concentration for determination of fold activation studies (40).

**Figure 2.**
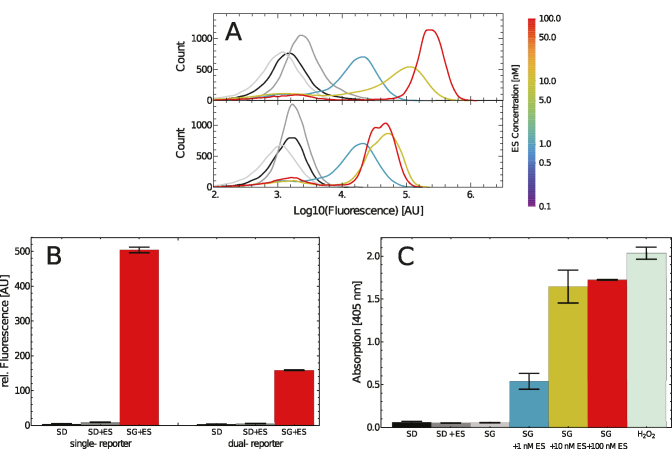
(**A**) Dose–response histograms of the single-reporter system (top) and the dual reporter system (bottom). Black histogram: SD only, Dark gray histogram: SD + 100 nM ES, Light gray histogram: SG only. (**B**) Fold-activation histograms of Venus and GOase-tGFP reporter systems obtained by computing the population mean of the corresponding fluorescence histograms: SD -glucose only; SD + ES - glucose and 100 nM ES; SG+ES - galactose and 100 nM ES. Fold inductions of 111 (single reporter) and 99 (dual reporter) were observed. (**C**) ABTS assay for determination of GOase activity in cell supernatants upon induction of gene expression at varying ES concentrations. H_2_O_2_: positive control with 0.25 % H_2_O_2_ added. The error bars indicate the standard deviation of the biological triplicates, except for (C, SG + 100nM ES) where only a duplicate was available.

To investigate the dose-dependent switching behavior, both reporter systems were induced with an ES concentration between 0.1 and 100 nM respectively, in presence or absence of galactose. With increasing concentrations of the inducer ES an increase of the overall cellular fluorescence was observed by flow cytometry which indicated a dose-dependent fluorescent protein expression for both reporter types (Figure 2A and Supplementary Figure S7).

Interestingly, with the double reporter system maximum cellular fluorescence was reached at a lower ES concentration (20 nM) compared to the single reporter (35 nM) (Figure 2B and Supplementary Figure S7), whereas the mean fluorescence signal was weaker. This effect can probably be attributed to the different fluorophore properties, but at least partially may also be caused by the T2A setup.

As a result, for growth in presence of ES or galactose only, virtually neither Venus, nor tGFP, nor GOase reporter gene expression (Figure 2C) was detected. Hence, the AND gate is tightly regulated and we were able to annihilate the *GAL10* leakiness by ES dependent induction of LexA-ES-AD that controls MCP-VP64 synthesis. Dose-dependent galactose switching could be demonstrated but resulted in a fluorescence distribution of Venus producing cells over a broad range rather than a distinct off/on switch (Supplementary Figure S8 and Supplementary Section SI3.2.1). Hence, switching with galactose was not further investigated.

To determine, whether active GOase is secreted in the dual reporter setting and whether tGFP-mediated cellular fluorescence correlates with enzyme activity, a comparative ABTS assay for cell pellet and supernatant was performed (Supplementary Figure S9A and B). *S. cerevisiae* is known for relatively low secretion levels (41), therefore the supernatant of 1 l of induced cell culture was concentrated and the cell pellet was disrupted. The expression of GOase was checked by SDS-PAGE and enzyme activity by ABTS assay. As expected, GOase was shown to be mostly present and active in the cell supernatant. No translational readthrough of the vNAR-T2A-tGFP was observed (Supplementary Figure S9C) corroborating the notion that ribosomal skipping efficiently works in *S. cerevisiae* (36).

Cell growth was occasionally reduced for the GOase-tGFP system for high ES concentrations, indicating that the metabolic burden is slightly increased by high protein expression and H_2_O_2_ production by GOase. This may also be the reason for the observed decrease of GOase activity upon high level expression (Supplementary Figure S10).

To investigate the potential detrimental effects of H_2_O_2_ that is generated by the conversion of galactose by GOase, we performed a serial dilution spotting assay at varying inducer concentrations. As shown in Supplementary Figure S11, the growth and/or induction media seems not to influence cell viability (see Supplementary Section SI3.2.2.)

### A fine adjustable AND gate with predictable switching behavior

It was recently suggested (42), that instead of the fold activity, the quality of a switch should rather be determined by a detailed analysis using receiver-operator-characteristics (ROC) curves.

Since some of our dose–response curves are bimodal, the fold activation that only accounts for the bulk behavior is only a rough measure of switching performance. We hence used ROC curves that incorporate the cell-to-cell variability and give thus a better estimate of the device’s functionality at single-cell level.

For a perfect switch design the fluorescence histograms of the ON and OFF state are well separated. It is hence possible to define a threshold parameter *T* that classifies all cells as being in the ON and OFF state, that show a fluorescence value above and below this threshold. For each possible value of *T*, the ROC curve indicates the fraction of ON cells that are correctly classified (true positive) versus the fraction of cells that are falsely classified (false positives). For a dysfunctional switch device where cells in the ON and OFF state can not be distinguished, the ROC curves is a diagonal line, representing for each threshold the same false positive and false negative rate. The better the separation between ON and OFF state, the more the ROC curve transforms toward a step-function jumping from point (0,0) to (0,1) and then staying constant (detailed explanation of the math in the Supplementary Section SI3.3).

**Figure 3.**
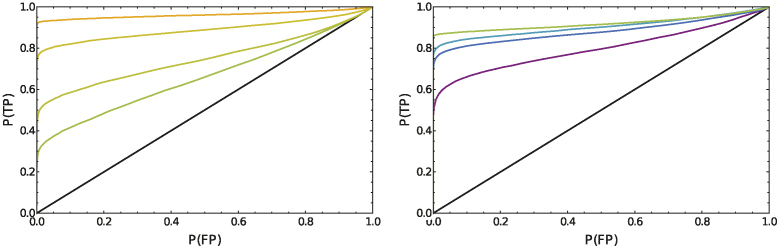
(Left) ROC curves for the single-reporter system. The black line indicates the diagonal ROC curve that would result from a dysfunctional switch. The colored lines correspond to the single-reporter system induced with β-ES concentrations of 1 to 20 nM. The curves should be read like this: If one seeks for the 1 nM system a false-positive rate of 50%, this would result in a true-positive rate of ∼60%. By shifting the threshold, one can archive a false-positive rate of 10%, but this comes along with a decrease of the true-positive rate to 40%. (right) ROC curves for the dual-reporter system. The black line indicates the diagonal ROC curve that would result from a dysfunctional switch. The colored lines correspond to the single-reporter system induced with β-ES concentrations of 0.1 to 5 nM. Due to the high sensitivity of the system, the ROC does not change very much above concentrations of 0.5 nM.

The ROC curves for the single reporter and dual reporter system are given in Figure 3 and indicate that, upon increasing the ES concentration, both reporter systems operate as switches with well defined and separated states. The noticeable gap between the curves and the true-positive rate of 100% originates in the small but mostly unavoidable fraction of cells that do not switch at all.

Further experiments were performed to obtain a better understanding of the AND gate dose dependency. For the Venus reporter system fluorescence could be detected beginning at 5 nM ES and a maximum fluorescence was reached at ∼35 nM ES. Due to the increased sensitivity of the GOase-tGFP dual reporter system, the ES concentration resolution was refined at lower concentrations. Fluorescence increase could already be measured at 0.1 nM ES reaching a maximum value at 20 nM concentration (Supplementary Figure S7). To evaluate which underlying principles account for the observed dose dependence, we developed a minimalistic dynamical mathematical model. The model comprises three important processes, namely the diffusion of the transcription factor (TF), the expression of mRNA regulated by the TF, as well as degradation of the fluorescent reporter protein (the full model is described in the ‘Materials and Methods’ section and Supplementary Section SI3.4).

We asked why the fluorescence distribution shows the observed bimodal behavior that can not be observed in our deterministic model. It is well known that stochasticity in small systems or at low concentrations can give rise to some effects, that are not covered by the deterministic description (43–45). Our switch is functional at concentrations in the range of 1 to 10 nM in a small system (nucleus size of yeast ∼3 μm^3^ (46)), thus it is necessary to consider stochastic effects. To this end, adopting the parameters obtained by fitting of the deterministic model, we built a stochastic model (detailed data handling described in Supplementary Section SI3.5). Our model can be understood as a modification of the three-stage gene expression model analyzed by Shahrezaei *et al.* (47), where we replaced the switchable by an inducible promoter.

**Figure 4.**
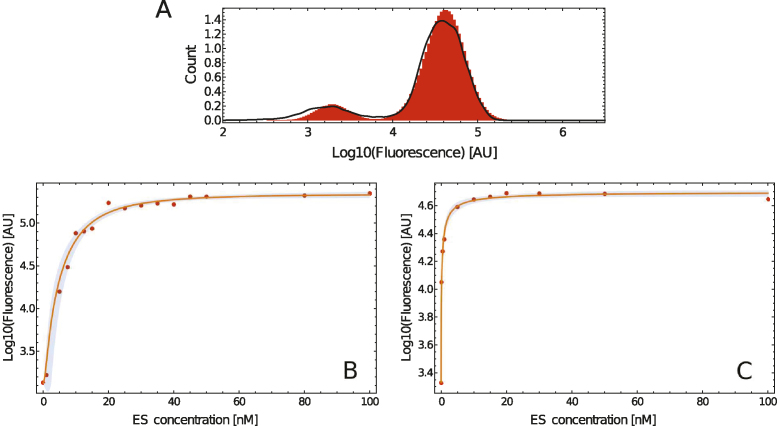
(**A**) Comparison of the stochastic model (histogram) with the experiment (solid line) for the dual-reporter system at an ES concentration of 5 nM. A detailed comparison for both reporter systems and various concentrations are given in Supplementary Figure S15. (**B**) Dose–response curve of the single reporter system. Markers indicate experimental results, the solid line indicates the expectation according to our mathematical model. The blue band denotes the 90% confidence interval of the mean of the model. The standard-error bars of the experimental data are smaller than the markers. (**C**) Dose–response curve of the dual reporter system. Markers indicate experimental results, the solid line indicates the expectation according to our mathematical model. The blue band denotes the 90% confidence interval of the mean of the model. The standard-error bars of the experimental data are smaller than the markers.

An exemplary comparison between the experimental data and the data obtained from the stochastic model are given in Figure 4A and Supplementary Section SI3.5. The stochastic model qualitatively reproduces the distributions observed in the experiments. This indicates that our model captures the most important underlying principles as well as that the diffusion of ES is the origin of the bi-modality. In this context, it is important to note that our model is by purpose not designed as a complex model that tries to capture all ongoing processes in full details. The intention of the model is rather to describe universal principles that can lead to the observations.

Figure 4B and C show the mean of the dose–response histograms together with the fitted model. Using our computational model that accurately predicts the fluorescence values of the two reporter systems, one can now use the model to obtain further results *in silico*. In particular, it is possible to exactly quantify the ES concentration that is necessary to reach a desired fluorescence.

In conclusion, the AND gate in principle enables the fine adjustable expression of different GOI at low nanomolar ES concentration. Outside this rather small range GOI expression is broadly binary.

### Time dependent AND switching

**Figure 5.**
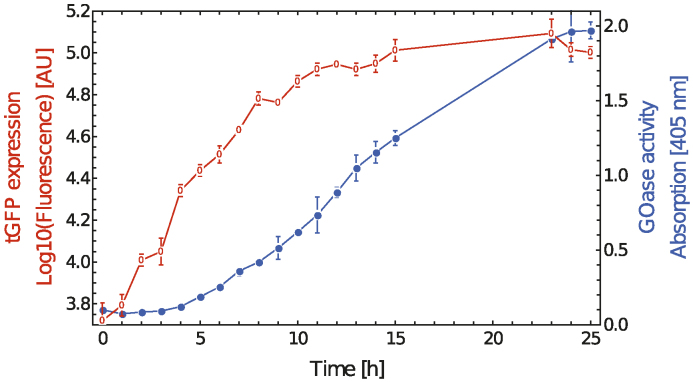
(Orange) Time-dependent measurement of gene expression for the dual reporter system. Induction was performed with 100 nM ES and galactose. (blue) Time-dependent measurement of GOA activity examined by ABTS assay. The error bars indicate the standard deviation of the measurement means.

For both systems flow cytometry analysis was performed on cells incubated with 10 nM ES and galactose (Supplementary Figure S12). Additionally, for the double reporter system an incubation using 100 nM ES was performed (Figure 5).

A first increase of fluorescence was measured after 6 h for the Venus reporter system (Supplementary Figure S12) and after 2 h of induction for the GOase-tGFP reporter system (Figure 5). After 15 h both systems seemed to be fully activated and no further increase of the fluorescence was detected.

For the double reporter system GOase activity was determined by ABTS assay (Figure 5). No activity was detected from hour 1 to 5. After 6 h first GOase activity was observed, which continued to increase until the hour 23.

As expected, GOase activity correlated well with cellular fluorescence, though measurement of GOase activity was delayed for a couple of hours (Figure 5). Different synthesis pathways (cytoplasmic expression versus secretion) may account for these differences. Nevertheless, our data indicate that co-translation via T2A ribosomal skipping can be used as a tool to follow time-dependent expression of a GOI and to quantitate its accumulation.

**Figure 6.**
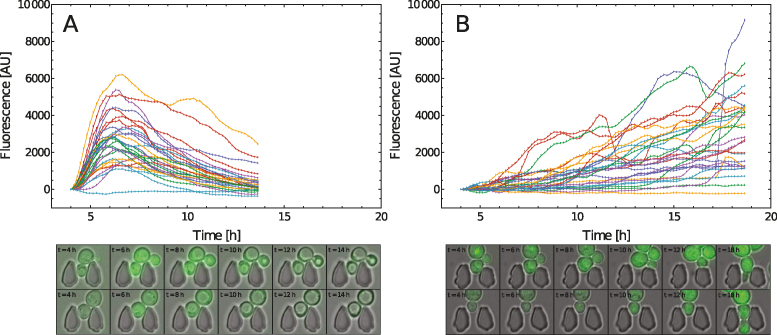
Single-cell traces from 30 cells for 100 (**A**) and 20 nM ES (**B**), respectively; Corresponding fluorescence microscope imagery of selected cells for given time-points, the cells are trapped in micro-patterned traps in continuous flow.

Flow cytometry provided snapshots into the distribution of fluorescence across a population of cells. To gain a deeper insight into gate dynamics of individual cells, time-lapse measurements were recorded for the dual reporter system. Single-cells immobilized in a controlled environment on a microfluidic chip were exposed to a continuous flow of induction media at concentrations of 100 and 20 nM ES, respectively (Figure 6A and B).

Distinctive differences are observed in the single-cell traces for the two ES concentrations chosen. At nominally high concentrations of 100 nM, the cells exhibit homogeneous dynamics without any phase shift, i.e. the cells attain maximum fluorescence within a short temporal window at ∼7 h post induction. The cells completely arrest their growth at *t* = 12 h (Figure 6, left), with no budding events detected after this time point. This is likely the result of a combination of detrimental effects, such as metabolic overload and potential toxicity GOase-mediated generation of H_2_O_2_ and requires further investigation (beyond the scope of this study). In this arrested state, the cells no longer produce tGFP and the subsequent decay in fluorescence is deemed to stem from photo bleaching and potentially also bleaching due to presence of protein damaging H_2_O_2_.

For the lower induction concentration of 20 nM ES (Figure 6, right) the cells behave more heterogeneously. Some remain switched off and those that switch on, do so with an intermittent phase shift. Unlike in the high concentration case, some traces indicate oscillations with cells turning off and on. Similarly to the higher ES concentration test, a portion of the cells exhibit an arrested state, however, another portion of the cells bud continuously throughout the experiment and exhibit high levels of fluorescence.

## DISCUSSION

### scRNA CRISPR/dCas9 platform enables construction of complex regulatory circuits

A wide range of technologies have recently emerged for editing and manipulating genetic circuits (48–50). Ideally, an universal, robust and adaptive toolbox for generation of gates and circuits should be available. Toward this end, we designed a logic AND gate in *S. cerevisiae* that is based on dCas9-mediated transcription activation (10,51,52). Recently, transcription activation with scRNAs addressing a row of *tet*O recognition sequences placed in front of a GOI via recruitment of a transcription activator (e.g. MCP-VP64) has been proven as a versatile tool for the induction of gene expression at a high level (6,7). However, particularly with increasing the number of *tet*O boxes an elevated level of basal transcription was observed that was mainly attributed to leakiness of galactose-induced dCas9 expression, since the dCas9 coding sequence was placed under *GAL10* promoter control (10,32). Moreover using galactose as an inducer of gene expression fine tuning of target gene expression is not possible (53). To overcome this problem, we combined the inducible expression of dCas9 with an ES-dependent expression of the MCP-VP64 transcription activator. Experimental results with mathematic modeling and microfluidic single cell measurements supported the finding of a tightly regulated AND gate with no measurable basal transcription activation.

Using the scRNA CRISPR/dCas9 system as a platform for AND gate construction, we observed high levels of reporter gene expression levels by recruitment of several scRNAs to a tandem array of 7 *tet*O boxes (Supplementary Figure S3). Moreover, since the scRNA was constructed such that it contains two MCP recognition sequences (9,10), two MCP-VP64 molecules can simultaneously bind to one scRNA molecule. This is corroborated by the finding that the reporter gene expression level via dCas9 scRNA and MCP-VP64 combination is much higher compared to TetR-VP16 recruitment (Supplementary Figure S3) (4).

Moreover, the β-estradiol inducible so called LexA-ER-AD expression system should allow the tunable expression of a target gene. The estrogenic hormone β-estradiol is a versatile input for the regulation of heterologous transcription factors, since it has been reported to ensure tight regulation upon binding to the hormone-binding domain of the human estrogen receptor (ED) that also works in yeast. In the absence of inducer, the ED interacts with the Hsp90 chaperone complex which results in sequestering the protein to the cytoplasm. Introduction of β-estradiol displaces Hsp90 and the ATF translocates to the nucleus, where it binds to its cognate DNA (*lexA* boxes) and activates transcription (Supplementary Figure S1) (12,13,32).

Several activator domains were tested in the LexA-ER-AD context, such as B42, B112, VP16 and GAL4 AD. Ottoz *et al.* reported that in comparison the AD B112 showed higher fold activation than the AD B42 and compared to VP16 and Gal4AD displayed less steep titration curves. Hence, we chose B112 as activation domain (2). Moreover it was shown not to disturb cell growth. Also in the context of MCP-VP64 expression, we did not see any negative impact on cell viability (Supplementary Figures S5 and S11).

### An AND gate design for the expression of various genes

To demonstrate, that our AND gate works well for different genes of interest, we showed the functionality not only with a single fluorophore reporter system, but also with a more complex dual reporter. The dual reporter consisted of the oxidoreductase GOase and the fluorophore tGFP combined by a T2A peptide. Since the tGFP sequence was placed behind the GOase gene, full-length translation of GOase and T2A peptide are required to enable tGFP formation upon ribosomal skipping (36). 2A oligopeptide sequences mediate a translational recoding event in a way that the elongating ribosome when encountering the 2A sequence skips the formation of a specific glycylprolyl peptide bond thus allowing for the synthesis of two proteins from a single open reading frame (54–56).

We and others have shown that fluorophore formation provides an easy way to use tGFP fluorescence to verify correct expression of a GOI in *S. cerevisiae* (36,55,57). We chose GOase as a model reporter protein, since it allows for the simultanous quantitation of enzyme accumulation using a simple activity assay that is based on peroxidase-mediated oxidation of ABTS upon GOase-mediated formation of hydrogen peroxide. ES dependent expression of secreted GOase shows that our AND gate/dual reporter system can be used to indirectly monitor expression of a GOI via following concommitant tGFP expression (see Figure 5 and Supplementary Figure S12B). We found a correlation between tGFP accumulation in the yeast cytoplasm and accumulation of H_2_O_2_ producing enzyme in the concentrated culture supernatant corroborating the notion that *S. cerevisiae* is known to display relatively low levels of accumulation of secreted proteins (41,58). Likewise, a construct, where the GOase moiety was replaced by a shark-derived antibody domain displayed ES-dependent expression (Supplementary Figure S9C).

### Both reporter systems are time and dose dependent

Our AND gate was constructed not only to achieve a high fold activation, but also to enable precise adjustment of reporter gene expression levels. We therefore measured reporter gene expression for increasing ES concentrations. High levels of the reporter genes were observed with relatively low inducer concentration. For the Venus reporter system 35 nM ES and for the GOase-tGFP reporter system 20 nM ES were sufficient to reach the maximum level of fluorophore expression. A reproducible dose dependency was observed and can be predicted by our model, thus allowing one to modulate target gene expression in our AND gate by controlling the ES concentration (Figure 2 and Supplementary Figure S7). In both cases and in accordance with the model, the concentration range of inducer ES, in which the reporter systems showed dose dependency was relatively small. Outside this range the reporter gene expression was either ON or OFF and therefore showed binary behavior.

To demonstrate that our experimental results are consistent with the understanding, that ES mediates the diffusion of the transcription factor, we developed a minimal mathematical model. Although only based on two differential equations, we were able to correctly predict the dose dependent fluorescence values for both reporter system. One interesting observation in our experiments is the bimodal fluorescence distribution that can not be detected in the deterministic model. Hence, to correctly account for the stochastic effects, we additionally built a stochastic model. Herewith and based on the results of the deterministic fitting we could qualitatively rebuild the full fluorescence distributions for both reporter systems at different ES concentrations.

We further investigated the time-related switching behavior upon addition of galactose and 10/100 nM ES. Even if there were small differences between the two reporters, the AND gate needed roughly 2 h until first reporter gene expression was detectable and 15 h to be fully activated (Figure 5). Over the time a graduated ON switching was detected.

In this context, it must be noted that high ES concentrations influenced the expression of GOase and to a lower amount of tGFP. We do not see any influence for the single reporter system. Hence, we assume that this is related to the synthesis of active GOase and accumulation of the cytotoxic compound H_2_O_2_ due to the presence of GOase substrate galactose in the culture medium.

### Single cell analysis of detrimental effects on cell growth

To achieve a better understanding of the switching dynamics and the potential temporal influence on the systems, time dependent measurements were performed. While the population data and single-cell traces (Figure 6) are not directly comparable, some agreement between the recorded dynamics are found. The time until a significant increase of fluorescence is recorded is ∼5–7 h post induction for both cases. Further comparison between population data and single-cell traces are hindered by properties inherent to the measurement methods. Population data measured over time spans larger than the doubling time of the organism may incur a bias. In this case the measurement biases toward fluorescent cells which are observed to bud more frequently than less fluorescent ones.

The observation of a state of arrested cell growth in the time-lapse single-cell analysis is evidence of detrimental effects on cell growth for the dual-reporter system. This effect appears to be dose dependent, as it affected all cells at 100 nM, yet only a portion of the cells at 20 nM ES. It may be a key limiting factor in GOase protein production with this system, also on the population level. While there is no conclusive evidence as to a single cause of this detrimental effect, it is deemed to be a combination of metabolic overload, increased sensitivity of the system to ES and also the production of potentially toxic H_2_O_2_.

We cannot exclude that this observation may also be a result of the different growth conditions in the microfluidic device, since ES-dependent cell growth arrest was not detected upon bulk cell cultivation in flasks. Nevertheless, for better accordance with reality, we incorporated detrimental effects into the stochastic model. We observe that these detrimental effects depend on the ES concentration in a hill-function like manner.

### Applications: adapt the AND gate to the requirements

By combining the different components to an AND gate we achieved a robust, predictable and tightly regulated platform for expression of multiple target genes with a tunable fold activation. There are many possibilities how to adapt the system to other needs, most of them on the genetic level. One possible approach to modify the maximum fold activation would be to change the number of *tet*O-binding sites or *lexA* boxes. Both proved to effect the expression level (2,10). Moreover, one may change the transcription activator of LexA-ER-X or MS2-X to a more or less potent one. In fact, the functionality of the whole system could be converted to an repressor system simply by changing the VP64 into an repressor like KRAB (59,60). It would also be pretty easy to add scRNAs binding to other sequence(s) and therefore activate multiple target expression simultaneously (10,61).

## Supplementary Material

Supplementary Data
