# Supplementary material for: A tightly regulated and adjustable CRISPR-dCas9 based AND gate in yeast
Source: Nucleic Acids Res. 2018 Nov 22;47(1):509–20. doi: 10.1093/nar/gky1191 (PMC6326796; doi:10.1093/nar/gky1191)
Supplement: Supplementary Data [file gky1191_supplemental_files.pdf]

Supplementary Data

A tightly regulated and finely adjustable CRISPR-dCas9 based AND gate in yeast

Anja Hofmann, Johannes Falk, Tim Prangemeier, Dominic Happel, Adrian Köber, Andreas Christmann, Heinz Koeppl and Harald Kolmar

1 Supplementary Figures

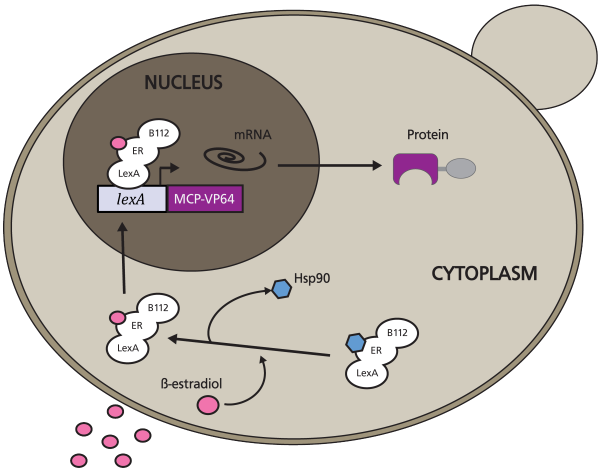

Figure S1: Induction of LexA-ER-B112 fusion protein transcription activation by ES. The fusion protein containing the LexA binding domain, the human estrogenic receptor ER and the transcription activator B112 are bound to Hsp90 in absence of ES. After addition of ES Hsp90 is displaced and the complex now can enter the nucleus and bind to the *lex*A boxes preceding the MCP-VP64 gene. The gene is transcribed to mRNA, tranists from the nucleus to the cytoplasm and is translated into a functional fusion protein.

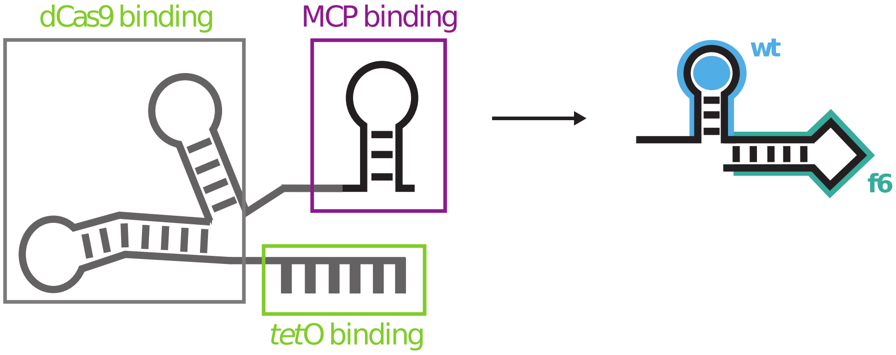

Figure S2: *scRNA design.* The scRNA contains a 20 bp region for the specific targeting of *tet*O in front of a target gene, two loops designed for dCas9 recruitment and two loops for MCP binding (MS2, f6).

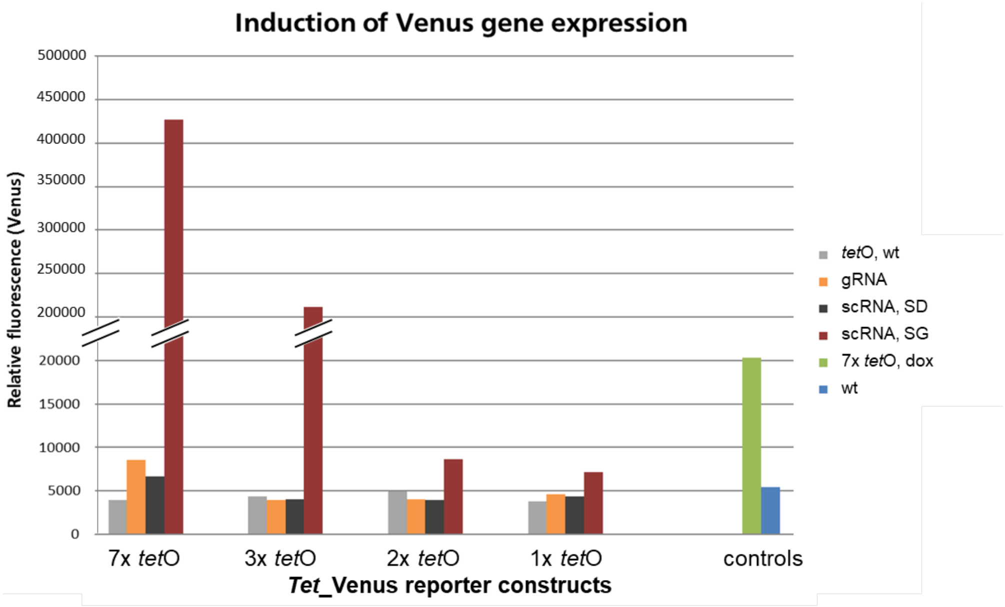

Figure S3: Induction of Venus expression using different tandem copies of *tet*O. Clones containing 1, 2 3 or 7 copies numbers of *tet* operator sequences respectively in front of the reporter gene Venus were designed after Zalatan *et al.* [1]. The *tet*O wt strain contained no CRISPRi genes, but the *tet*O reporter constructs. The samples called gRNA contained an uninducible system with a direct VP64 fusion to dCas9. The strains containing the MCP-VP64 fusion were called scRNA, SD for uninduced and scRNA, SG for galactose induced samples. The controls display the results for the 7x *tet*O Venus strain that contains a genomically integrated rtTA (see Supplementary Table S1) induced with doxycycline (SD) [1] and the wild type wt, which contained no genes for Venus expression.

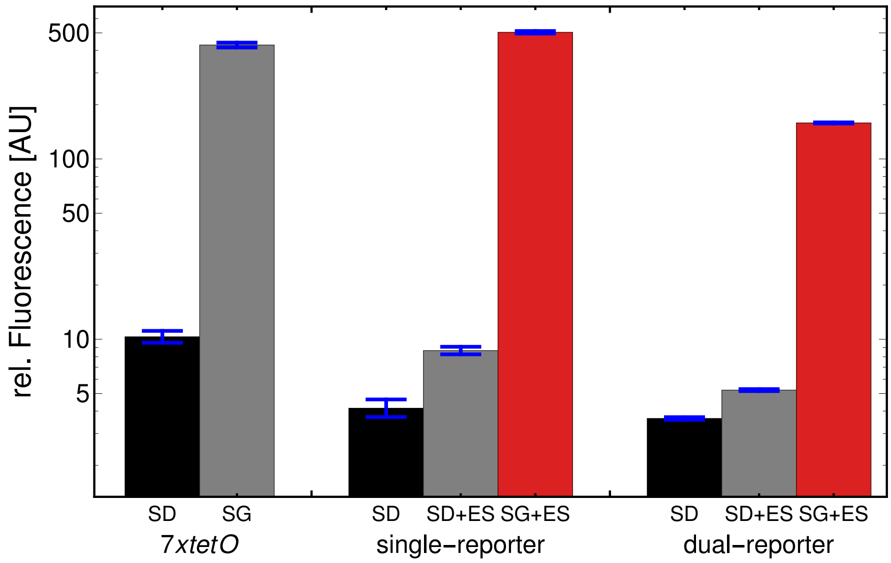

Figure S4: Comparison of the basal expression of the pure SD/SG based system[1] and our single and dual-reporter system. For better comparison the y-axis is shown in log-scale. The error bars indicate the standard deviation of the biological triplicates.

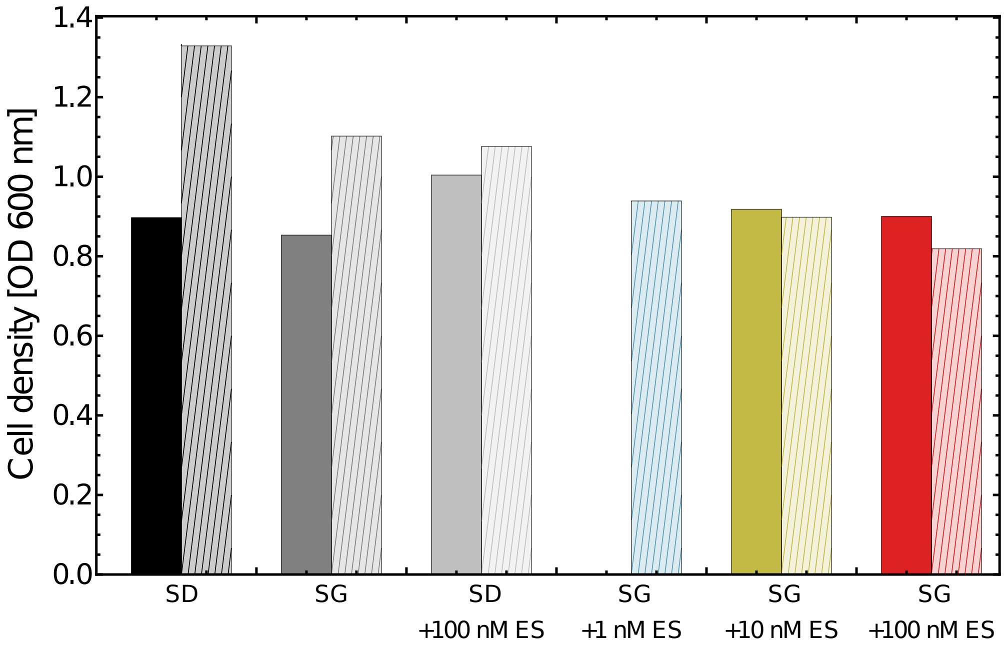

Figure S5: Influence of induction of reporter gene expression on cell growth. The strains were inoculated to an OD600 of 1 in SG medium and grown over night in SD medium, SG medium or induction medium containing SG and 1nM, 10 nM or 100 nM ES, respectively. For the Venus reporter system (filled) no significant influence in growth could be detected, whereas with increasing amount of ES the cell density of the GOase-tGFP reporter system (hatched) decreased slightly.

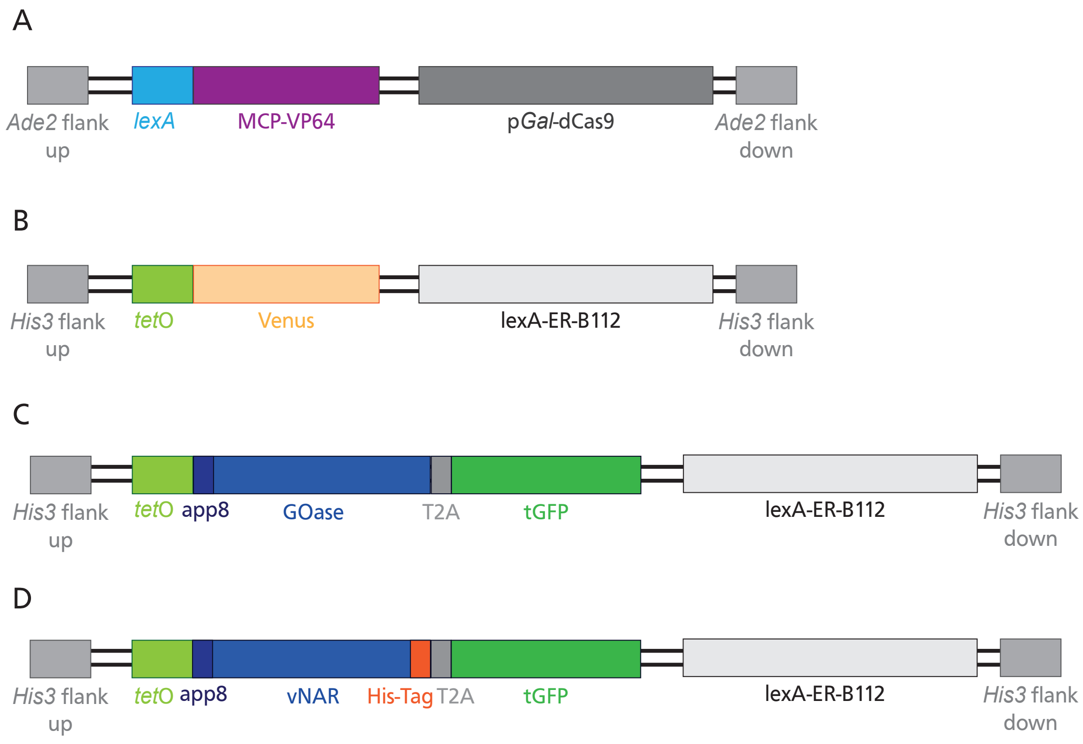

Figure S6: Schematic representation of genes inserted into the yeast genome by CasEMBLR. MCP-VP64 under control of 4 lexA boxes and dCas9 under control of pGal were integrated into *Ade2* locus (A). The constructs for the single (B), the double reporter system based on GOase-T2A-tGFP (C), as well as the additional one based on vNAR-T2A-tGFP (D) were integrated into the *His3* locus.

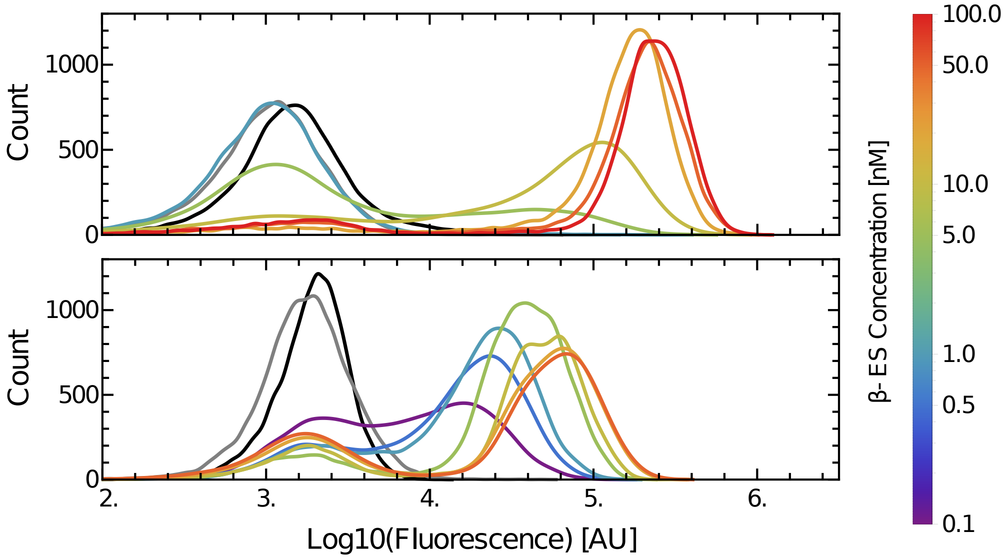

Figure S7: Dose-Response histograms of the single-reporter system (top) and the dual reporter system (bottom).The black histogram indicates the experiment where cells were grown in SD only, the gray histogram indicates SG without ES.

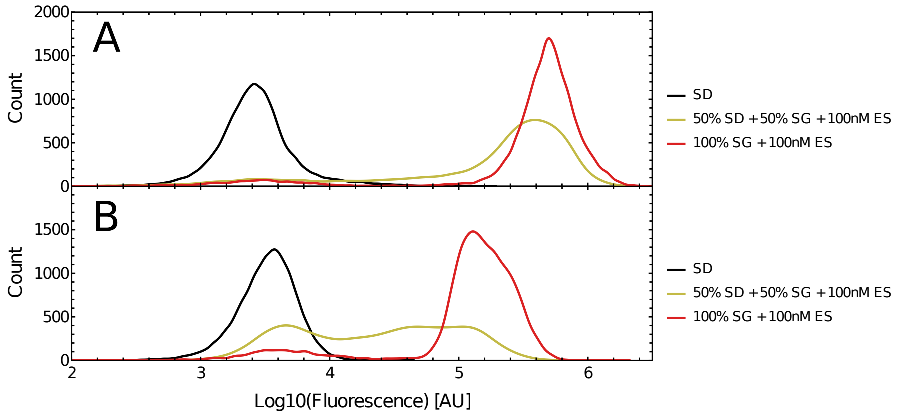

Figure S8: Test for Galactose tunability. The clones were induced with a constant amount of ES, but different SG concentrations. (A) single-reporter system; (B) dual-reporter system.

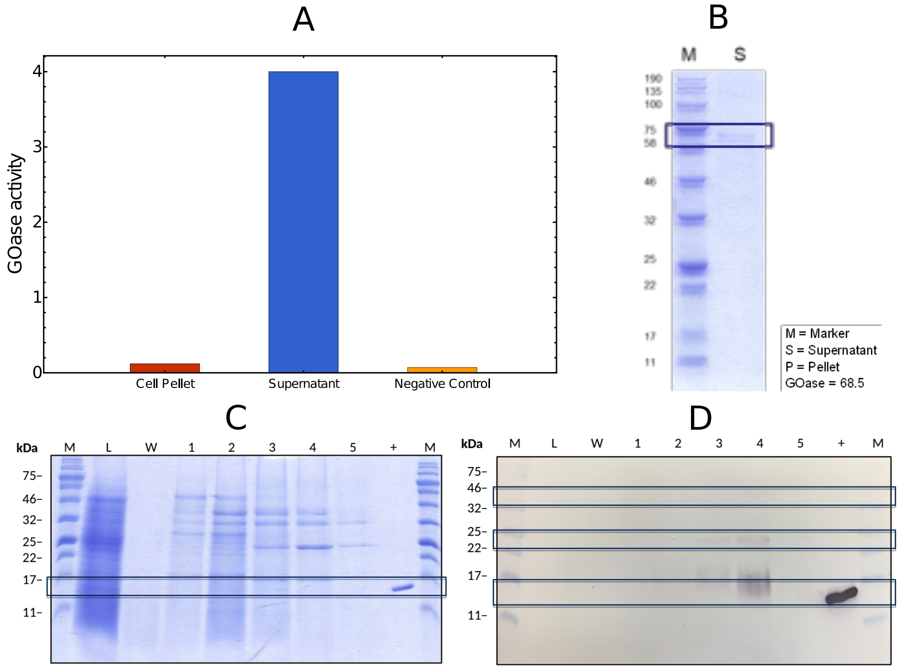

Figure S9: (A) Localisation of GOase activity. ABTS assay of the GOase-tGFP reporter strain after induction of 1 l of yeast culture. The supernatant was concentrated by cross-flow filtration with a Vivaflow 200, 10.000 MWCO Hydrosart (Sartorius) from 1 l to 50 ml. As negative control supernatant of uninduced cells was used. (B) SDS PAGE analysis of concentrated supernatant. (C) Purification of vNAR-T2A-tGFP reporter system cell lysate with immobilized metal ion affinity chromatography (IMAC). Samples were collected and are shown on a SDS page. (D) Western blot of samples shown in (C) immunostained with anti-His antibody (mouse) and anti-mouse IgG-AP (goat). For (C) and (D) is L=filtrated cell lysate, W=IMAC wash, 1-5=IMAC elution/peak fractions, +=positive control, vNAR His-tag. Expected sizes are vNAR=13.256 kDa, vNAR+app8=22.045 kDa, vNAR+T2A+tGFP=39,726 kDa, vNAR+app8+T2A+tGFP=48,512 kDa, tGFP=24,676 kDa. For SDS pages and Western blot Blue Prestained Protein Standard, Broad Range (11-190 kDa) from NEB was used.

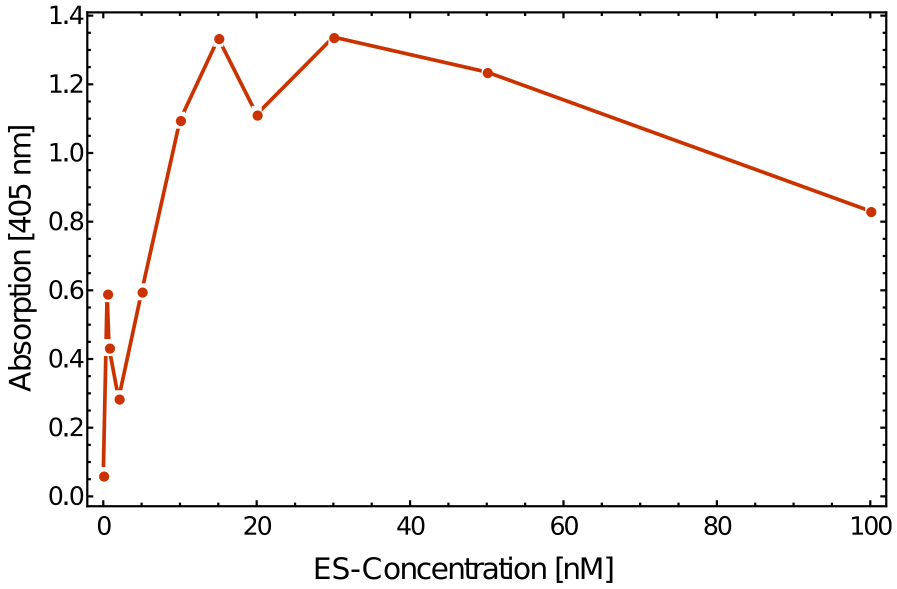

Figure S10: ABTS assay for dose dependent GOase activity.

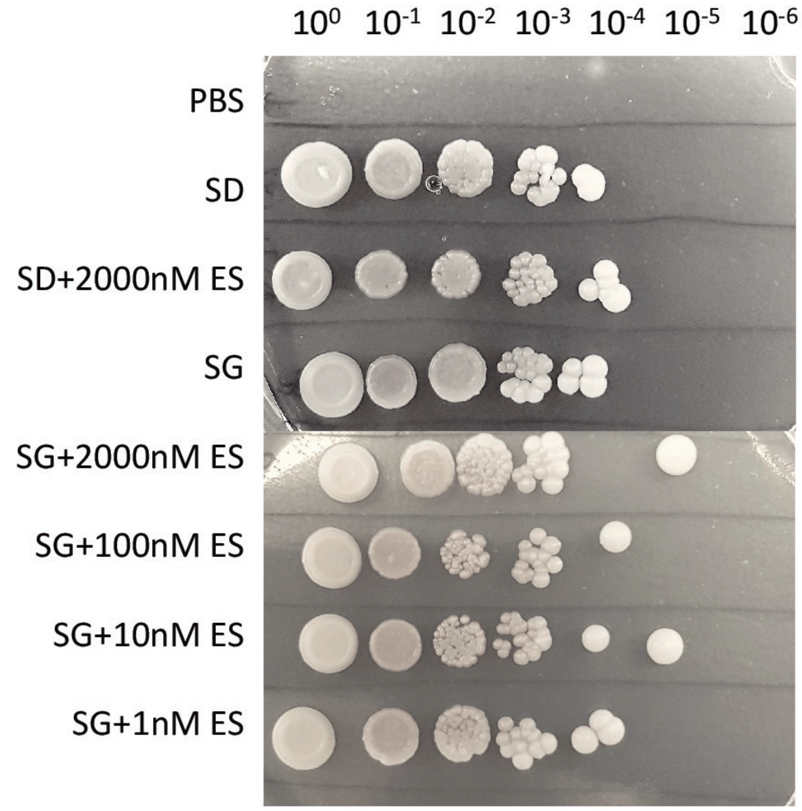

Figure S11: Serial dilution spotting assay or different concentrations of ES. The cells containing the GOase-tGFP reporter were grown in different media for 20 hours. Cells were separated from supernatant and suspended in PBS. A serial dilution was performed and 5 μL each were dropped on a SD-ura agar plate. The cells were incubated for three days.

A

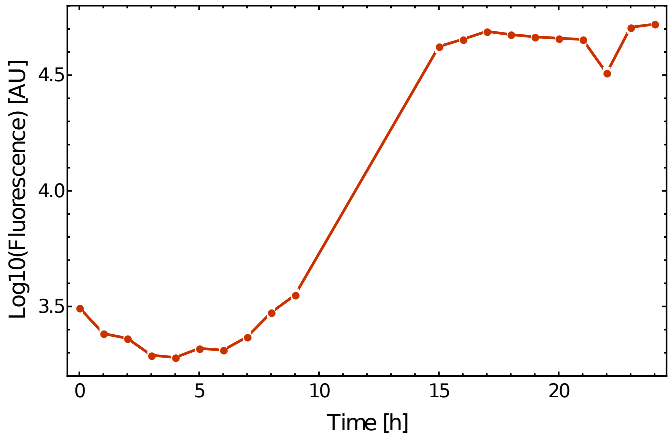

B

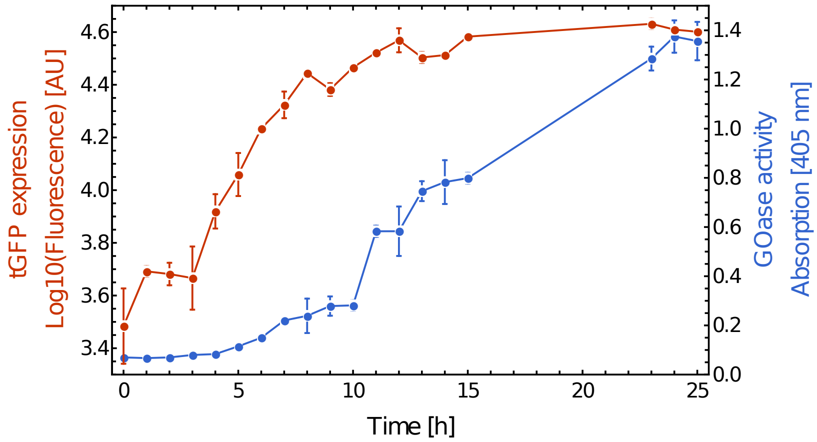

Figure S12: (A) Time dependent measurement of single reporter system. Induction was performed with 10 nM ES and galactose. (B) Time dependent measurement of gene expression for the dual reporter system. Induction was performed with 10 nM ES and galactose, (blue) time dependent measurement of GOA activity examined by ABTS assay, (red) time dependent measurement of tGFP by flow cytometry. The error bars indicate the standard deviation of the biological triplicates.

2 Supplementary Tables

Table S1: Strain constructs

Strain	Description	Genotype
SO992	W303 derivative	*MAT***a** *ura3 leu2 trp1 his3 can1R ade*
cSLQ.sc002	W303 rtTA-msn2	*SO992 HO::rtTA-msn2_hphR*
T2C	cSLQ.sc002 pTET07-Venus	*cSLQ.sc002 ade2::lexA-MCP-VP64_Gal10-dCas9 his3::TET07-Venus_lexA-ER-B112*
H2E	cSLQ.sc003 pTET07-app8-GOase-T2A-tGFP	*cSLQ.sc002 ade2::lexA-MCP-VP64_Gal10-dCas9 his3::TET07-app8-Goase-T2A-tGFP_lexA-ER-B112*
H4E	cSLQ.sc003 pTET07-app8-vNAR-His-Tag-T2A-tGFP	*cSLQ.sc002 ade2::lexA-MCP-VP64_Gal10-dCas9 his3::TET07-app8-vNAR-His-Tag-T2A-tGFP_lexA-ER-B112*
cSLQ.Sc003	cSLQ.sc002 pTET07-Venus	*cSLQ.Sc002 trp1::pTET07-Venus*
yJZC02	cSLQ.sc002 pTET01-Venus	*cSLQ.Sc002 trp1::pTET01-Venus*
yJZC03	cSLQ.sc002 pTET02-Venus	*cSLQ.Sc002 trp1::pTET02-Venus*
yJZC04	cSLQ.sc002 pTET03-Venus	*cSLQ.Sc002 trp1::pTET03-Venus*

Table S2: Plasmids for part amplification and strain construction

Nr.	Plasmid	GOI	Selection marker	Inducible	Addgene
1	p414-Cas9	Cas9	*trp1*	-	
2	pA6	gRNA ade2 + his3_2 (CASEMBLR)	*leu2*	-	
3	pJZC588	scRNA 2x (wt+f6) MS2	*ura3*	-	X
4	pFRP793	insul-(lexA-box)4-PminCYC1-Citrine-TCYC1	*ura3*	ES	X
5	pFRP880	PACT1(-1-520)-LexA-ER-haB112-TCYC1	*his3*	-	X
6	pJZC532	TET03-Venus	*trp1*	dox	X
7	pJZC625	scRNA 1x MS2 (Pol II prom, ribozyme-scRNA-ribozyme)	*ura3*	-	X
8	pJZC638	MCP-VP64, dCas9 [GAL]	*leu2*	gal	X
9	pSLQ1119	TETO7_Venus	*trp1*	dox	X
10	pYD_Leu_APP8-5005-2ap-GFP	app8 secretion signal, T2A	*trp1*	-	
11	pET22b	GOase	unknown	-	
12	pJZC518	dCas9	*leu2*	-	X
13	pJZC519	dCas9-VP64	*leu2*	-	X
14	pJZC523	gRNA for yeast cells	*ura3*	-	X
15	pJZC530	TET01-Venus	*trp1*	dox	
16	pJZC531	TET02-Venus	*trp1*	dox	
17	pJZC532	TET03-Venus	*trp1*	dox	
18	pSLQ1119	TETO7_Venus	*trp1*	dox	
19	pJZC638	MCP-VP64, dCas9 [GAL]	*leu2*	gal	X
20	pvNAR_intern	vNAR (anti-Matuzumab)	*trp1*	-	

Table S3: Oligonucleotides for part amplification

Name	Function	Sequence
Ade1	Ade flank_up2	GGCATACGATGGAAGAGGTAACTTCGTTGTAAAGAATAAGGA AATGATTCCGGAAGCTTTGGAAGTACTGAAGGATCGTC
Ade10	Ade flank_down2	AAATGAGCTTCAAATTGAGAAGTGACGCAAGCATCAATGGTA TAATGTCCAGAGTTGTGAGGCCTTGGGGCAATTTCGTT
Ade11	Ade flank-lexA_up1	GGAAGTACTGAAGGATCGTCCTTTGTACGCCGAAAAATGGGC ACCATTTACTAAAGAATTCGGCCAGTGAATTGTAATAC
Ade12	lexA_MCP_rv	TGGGCATCTCGAGAGACATTGTATATGAGAGATATCGAATTC CTGCAG
Ade13	MCP_lexA	GGATCCCCCGGGCTGCAGGAATTCGATATCTCTCATATACAA TGTCTCTC
Ade20	dCas9_Ade flank down1_rv	GGCCTTGGGGCAATTTCGTTAATAAGCAATTCCCCTGTTTCT AAATAGAACATTTCCACACCCACACAATTATAAGCAAA
His1	His flank_up2	CGATCTTCCCAGAAAAAGAGGCAGAAGCAGTAGCAGAACAGG CCACACAATCGCAAGTGATTAACGTCCACACAGGTATA
His3	His flank_7tetO_up1	TTAACGTCCACACAGGTATAGGGTTTCTGGACCATATGATAC ATGCTCTGGCCAAGCATTGGTACCCGGGCCCTTAATTCGC
His4	tetO_lexAFP_rv	CGCCAACCAAGTATTTCGGAGTGCCTGAACGCAAATTAAAGC CTTCGAGC
His5	lexAFP_tetO	GTTTTGGGACGCTCGAAGGCTTTAATTTGCtCCGAAATACTT GGTTGGCG
His6	lexAFP_His flank_down1	AAGGCGCAAATCCTGATCCAAACCTTTTTACTCCACGCACGG CCCCTAGGGCCTCTTTAAGAGTGTAAACTGCGAAcCTT
His7	His flank_down2	CGACAACTGCGTACGGCCTGTTCGAAAGATCTACCACCGCTC TGGAAAGTGCCTCATCCAAAGGCGCAAATCCTGATCCA
His16	tetO_app8_rv	AACTGCAGTAAAAAATTGAAGGAAATCTCATCTCGAGCATCC CGAATTGAT
His17	app8_tetO	AATTACCCGGATCAATTCGGGATGCTCGAGATGAGATTTCCT TCAATTTT
His18	app8_Goase_rv	CATATGACGACCTTCGATATGGCCGCTGCTTCTTTTATCGAG TTGATACCC
His19	Goase_app8	AAAGAAGAAGGGGTACAACTCGATAAAAGAAGCAGCGGCCAT ATCGAAGG
His20	Goase_tefT_rv	CTTGAAAACAAGAATCTTTTTATTGTCAGTCTGAGTAACGCG AATCGTCG
His21	tetT_Goase	AGTGTGGCTTCGACGATTCGCGTTACTCAGACTGACAATAAA AAGATTCT
His22	tetT_lexAFP_rv	GCGGTGGAGCTCCAATTCGCCCTATAGGCGCAGTATAGCGAC CAGCATTC
His23	lexAFP_tetT	AATCGTATGTGAATGCTGGTCGCTATACTGCGCCTATAGGGC GAATTGGA
His33	Goase_2AP_rv	GCCGCAGGTCAGCAGGCTGCCGCGGCCCTCCTGAGTAACGCG AATCGTCG
His34	2AP_Goase_fw	AGTGTGGCTTCGACGATTCGCGTTACTCAGGAGGGCCGCGGC AGCCTGCT
His35	term_lexAFP_rv	GCGGTGGAGCTCCAATTCGCCCTATAGGCGAATTCTCTTAGG ATTCGATT
His36	lexAFP_t_fw	GATGAATGTGAATCGAATCCTAAGAGAATTCGCCTATAGGGC GAATTGGA
His41	vNAR_His6_tag_rev	TGCCGCGGCCCTCGTGGTGGTGGTGGTGGTGTTTCACAGTCA GAATGGTCC
His42	His6_tag_tGFP_fw	TCTGACTGTGAAACACCACCACCACCACCACGAGGGCCGCGG CAGCCTGCT

Table S4: Oligonucleotides for colony PCR and sequencing

Name	Function	Sequence
Ade15	Colony_Ade genome	AGACGGTAATACTAGATGCTGA
Ade18	Colony_Ade Insert_rv_lexA	ACATACAGAGCACATGCTCT
His10	Colony_His_2 genome_fw	CCCGTTCCTCCATCTCTTTT
His11	Colony_His_2 genome_rv (paper)	TGCCAGGTATCGTTTGAACA
His12	Colony_His_2 Insert_rv	ACCTTCACCGGAGACAGAAA
His14	Colony_His_2 Insert_fw_1000bp	CGATCCAGTTGCTGAAAGAA
Ade25	Colony_Ade_1 Insert_fw_lexA	AGACTATATTTCTTTTCGAGCTCCCTAG
Ade26	Colony_Ade_2 Insert_fw_MCP	AGAAAAGAAAAGTGGGTAGTATGGCTTC
Ade27	Colony_Ade_2 Insert_rv_MCP	AGAAGCCATACTACCCACTTTTCTTTTC
Ade28	Colony_Ade_2 Insert_rv_Eno2t	TGCATTATGCAATAGACAGCACGAG
His43	Colony_His_6 Insert_fw	AGACACGCAAACACAAATACACACAC
His44	Colony_His Insert_rv_tGFP	ACCATTCAGGGTGCCGGTAATAC

3 Supplementary Information

3.1 Gating

In order to maintain comparability and comprehensibility, we followed a minimal gating strategy. We applied the following two gate to all of the data:

- To remove debris we excluded all events with a forward-scatter area (FSC-A) signal below an experiment specific threshold (Fig. S13(left)).
- To remove possible doublets and cell groups we used a forward-scatter height (FCS-H) vs. forward-scatter area (FSC-A) density plot and excluded the cells that did not follow the expected linear relation (Fig. S13(right)) [2].

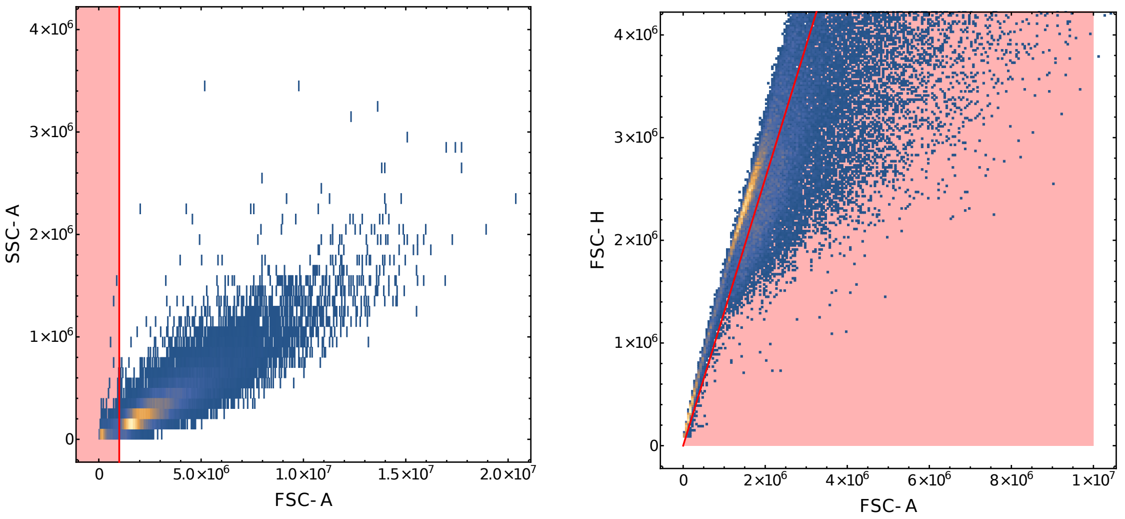

Figure S13: The two different gates that where applied to all the flow-cytometry data presented. The red areas are excluded. (left) Gate to exclude debris (right) Gate to exclude doublets.

3.2 Supplementary Methods

3.2.1 Stepwise galactose

Yeast cells were grown in SD-URA medium overnight at 30°C. The cell density was determined photometrically and $1 \cdot 10^7$ cells/mL were inoculated in synthetic complete medium leaking uracil containing different amounts of galactose (SG). Induction was completed by addition of 100 nM ES. Samples were analyzed after 20 hours of induction. Therefore, 300 μL of the cell culture were centrifuged at 8000 rpm in a Heraeus Biofuge Pico centrifuge to remove medium. Cells were resuspended in PBS and analyzed by flow cytometry followed as described in the main paper. Data analysis was performed using Mathematica 11 software.

3.2.2 Serial dilution spotting assay

The double reporter strain was grown over night in 5 mL SD-ura at 30°C. The cell density was determined photometrically and the suspension diluted to $1 \cdot 10^7$ cells/mL. Cells were induced in a total volume of 3 mL for 20 hours at 25°C. Samples for growth in SD-ura, SD + 2000 nM ES, SG -ura, SG + 2000 nM ES. SG + 100 nM ES, SG + 10 nM ES and SG + 1 nM ES were prepared. Following [3], 25 μL of the samples were transferred to a 96-well plate after induction and a serial dilution was perfomed in steirle PBS. 5 μL of each sample were dropped onto a SD- ura agar plate and cells were incubated at 30°C for up to three days to allow for growth differences to appear.

3.2.3 Stepwise galactose

Yeast cells were grown in SD-URA medium overnight at 30°C. The cell density was determined photometrically and $1 \cdot 10^7$ cells/mL were inoculated in synthetic complete medium leaking uracil containing different amounts of galactose (SG). Induction was completed by addition of 100 nM ES. Samples were analyzed after 20 hours of induction. Therefore, 300 μL of the cell culture were centrifuged at 8000 rpm in a Heraeus Biofuge Pico centrifuge to remove medium. Cells were resuspended in PBS and analyzed by flow cytometry followed as described in the main paper. Data analysis was performed using Mathematica 11 softwaremove medium. Cells were resuspended in PBS and analyzed by flow cytometry followed as described in the main paper. Data analysis was performed using Mathematica 11 software.

3.2.4 Construction, preparaion and detection and preparation of vNAR reporter strain

The strain containing an additional double reporter system is based on a vNAR-T2A-tGFP reporter construct. Cells were constructed as described in the main paper, whereby an exemplary anti-Matuzumab vNAR [4] gene was integrated instead of the GOase gene. The vNAR gene was designed with an additional a C-terminal His-Tag to allow purification via immobilized metal affinity chromatography (IMAC) followed by validation through immunostaining. After construction and verification of correct genomic integration, induction was performed similar to the GOase construct as described in the main paper.

Cells were grown overnight in 50 mL flasks in SD-URA medium at 30°C. After growth, cells were inoculated to a cell density of $1 \cdot 10^7$ cells/mL into 500 mL SG-URA and induction was completed by addition of 500 μL of a1 mM ES solution. Induction was performed at 30°C for 20 hours and vNAR-tGFP expression was verified by flow cytometry (data not shown). The cells were precipitated and the supernatant was concentrated to 3 mL using Amicon Ultra-15 Centrifugal Filter Units (MWCO 3 kDa, Merck Millipore) (data not shown).

The cell pellet was suspended in IMAC buffer A (10 mM imidazole), disrupted using a cell disrupter (Constant systems LTD) and cell debris was removed by centrifugation. For isolation of the His-tagged vNAR by IMAC, 30 mL cell lysate were applied to a 1

mL HisTrap HP column (GE). After washing with 10 column volumes (CV) buffer A, elution was performed with a linear gradient 0–100% buffer B (1 M imidzole) over 20 CV while collecting 1 mL fractions.

After SDS-PAGE (performed as described in the main paper) of selected samples, gels were either stained with Coomassie Blue for visualization of the whole protein content or blotted onto nitrocellulose membrane (Semi-dry Western Blot) for later immunostaining. 5 μL of the Blue Prestained Protein Standard, Broad Range (11-190 kDa) from NEB were utilized as marker. For each sample 15 μL were applied. Separated by washing steps (PBS with 0.05% Tween20) the membrane was blocked with 1.5% milk powder solution and incubated with monoclonal Penta-His antibody (produced in mouse, Quiagen) followed by Anti-Mouse IgG (whole molecule)–Alkaline Phosphatase antibody (produced in goat, Sigma Aldrich). Addition of NBT/BCIP substrate solution resulted in a purple-black precipitate in presence of alkaline phospotase activity enabling indirect detection of His-tagged proteins.

3.3 ROC-Curves

Our system represents a single-output logic gate that can be understood as a binary classifier. In signal detection theory, ROC (receiver operator characteristic) curves are used to characterize the performance of such classifiers, hence it was recently suggested to use ROC curves instead of the conventionally used fold-activation to display the gate's performance [5]. While the fold activation only accounts for the bulk behavior, the ROC curves incorporate the cell-to-cell variability and give thus a better estimate of the device's functionality at single-cell level. One important advantage of the ROC curve is that it characterizes the classifier performance independent of a particularly chosen threshold. Following [5] we obtain the coordinates $(x(T), y(T))$ of the points on the ROC curve for each possible fluorescence threshold T by the following two equations:

$$x(T) = \frac{1}{N} \sum_{i=1}^{N} \mathbb{1}(\bar{f}_i > T) \tag{1}$$

$$y(T) = \frac{1}{M} \sum_{i=1}^{M} \mathbb{1}(f_i > T) \tag{2}$$

where N and M are the number of cells in the OFF and ON state, respectively and f_i and $\bar{f}_i$ denote the corresponding fluorescence values of the ith cell.

3.4 Deterministic Model

We start with the given system of two differential equations:

$$\frac{\mathrm{d\,es_n}(t)}{\mathrm{d}t} = -\mathrm{es_n}(t)\,\gamma + \mathrm{es_m}(t)\,\gamma \tag{3}$$

$$\frac{\mathrm{dg}(t)}{\mathrm{d}t} = -\mathrm{g}(t)\,\delta + f(\mathrm{es_n}(t)) \tag{4}$$

$$\text{where: } f(\mathrm{es_n}) = \frac{\nu\ (\mathrm{es_n/k})^h}{1+(\mathrm{es_n/k})^h}$$

In the stationary state the time derivatives can be set to zero and one obtains:

$$es_n = es_m \tag{5}$$

$$g(\mathrm{es_n}) = \frac{f(es_n)}{\delta} = \frac{\phi\ (\mathrm{es_n/k})^h}{1+(\mathrm{es_n/k})^h} \tag{6}$$

where $\phi = \frac{\nu}{\delta}$. To be able to compare the model and the date, we introduce a scaling factor ρ that maps the number of g to the measured fluorescence value. Our fitting function is hence:

$$g(\mathrm{es_m}) = \frac{\Theta\ (\mathrm{es_m/k})^h}{1+(\mathrm{es_m/k})^h} \tag{7}$$

where $\Theta = \rho\phi = \frac{\rho\nu}{\delta}$ is now a generalized scaling factor.

Fitting this model to our experimental data we obtained the following parameter values:

Table S5: Single Reporter System

	Name	Estimate	Standard Error	Dimension
h	Hill coefficient	1.92834	0.33338	-
k	Michaelis-Menten constant	16.2291	1.75533	nM
Θ	Scaling factor	217235.	14049.1	AU

Table S6: Dual Reporter System

	Name	Estimate	Standard Error	Dimension
h	Hill coefficient	0.818458	0.139886	-
k	Michaelis-Menten constant	1.07945	0.248332	nM
Θ	Scaling factor	47708.6	2294.72	AU

3.5 Stochastic Model

Keeping in mind that the volume of the nucleus of a yeast cell is roughly $3 \cdot 10^{-15} L$, one notices that the Michaelis Menten constants obtained by the deterministic fit correspond

to $\approx 2 - 30 \frac{\text{ES particles}}{\text{nucleus}}$. In order to verify whether our model reproduces the bimodal distribution that was observed in the fluorescence histograms of the analyzed systems, we made hence use of a stochastic version of our model. The model is based on the chemical equations given in the paper:

$$\begin{aligned} \text{Diffusion: } \mathrm{ES_m} &\overset{\gamma}{\rightleftharpoons} \mathrm{ES_n} \\ \text{Expression: } \emptyset &\xrightarrow{f(\mathrm{es_n})} \mathrm{G} \\ \text{Degradation/Dilution: } \mathrm{G} &\xrightarrow{\delta} \emptyset \\ \text{where: } f(\mathrm{es_n}) &= \frac{\nu\ (\mathrm{es_n}/\mathrm{k})^h}{1 + (\mathrm{es_n}/\mathrm{k})^h} \end{aligned} \tag{8}$$

For the parameter values of k and h we used the values obtained in the deterministic model (see section 3.4 in this document). The concentration of ES_m is given by the experimental setup, the parameters ρ, δ and ν are constrained by the scaling factor Θ. γ was manually fitted for both systems separately. While γ should be independent of the GOI, the obtained values diver by a factor of ten. This might be due to some natural variation in the data, but could also be an indication that there are other reversible processes operating at slow time-scales.

Due to the used hill-function rates, our model does not have mass action-kinetics. It it was recently shown that this can lead to a loss of accuracy, especially of the higher order moments of the distribution [6, 7]. Nevertheless, since we later added extrinsic noise with a comparably large variance to the data (described below), the error due to the used nonlinear rates seems negligible.

In order to estimate the noise and fluorescence offset that is generated due to the auto fluorescence and the measurement device, we fitted Gaussian distributions to the negative control (only SD) for both reporter systems. The differences in the extrinsic noise distributions are mainly due to the different gain settings in the cytometer.

The final model-data was obtained as follows:

$$Log_{10}[S \cdot \rho + O] + N(0, \sigma^2); \tag{9}$$

where S are the raw data points obtained from the stochastic simulation of (8), ρ is the scaling factor constrained by Θ,ν and δ, O denotes the offset of the negative control and $N(0, \sigma^2)$ is a random variable distributed normally with mean 0 and variance σ^2 (see Fig. S14.

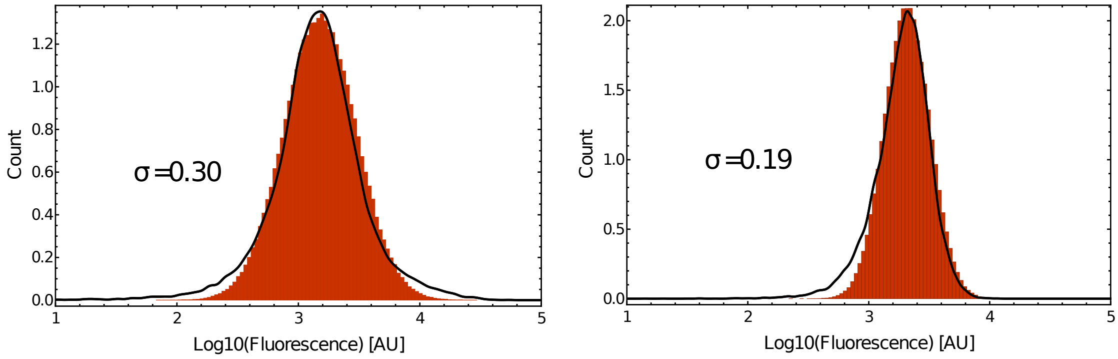

Figure S14: Distributions of the negative control to estimate the extrinsic noise distribution of the (left) single reporter and (right) dual reporter system. Solid lines are experimental date, histograms are generated Gaussian distributions. The σ indicates the fitted standard-deviation in linear scale. The offsets were obtained to be (left) 1194 [AU] and (right) 1943 [AU]

3.6 Detrimental effects

For the dual-reporter system we observed that most cells did show fluorescence as predicted by our model. Nevertheless, a small fraction of cells did not show any fluorescence signal above the auto-fluorescence value. In order to analyze the origin of this unexpected behavior, we included a generic detrimental effect into our model. Thereto, we defined the function $\zeta(ES)$ that depends on the ES-concentration and returns the fraction of cells that do not show any fluorescence. We set this fraction of randomly chosen data-points of our stochastic simulation to a value of 0 and rescaled the Θ parameter by $1/\zeta$ to keep the mean constant. We asked whether $\zeta(ES)$ is a constant function (and does not depend on ES) or whether there is a functional dependency of ζ on ES. While the statistics are not good enough to provide exact parameters, we can observe that the detrimental effects depend on ES in a hill-functional form. In Fig. S15(left), ζ was set to: $\zeta(ES) = 0.21\frac{1}{1+(6/ES)^{1.5}}$ with $[ES] = \text{nM}$.

The wrong predictions for 0.1nM do not indicate an inability of the model, but represent the high sensitivity of the system in this concentration regime. A small increase of ES by only 0.1ES results in large changes of the predicted distribution.

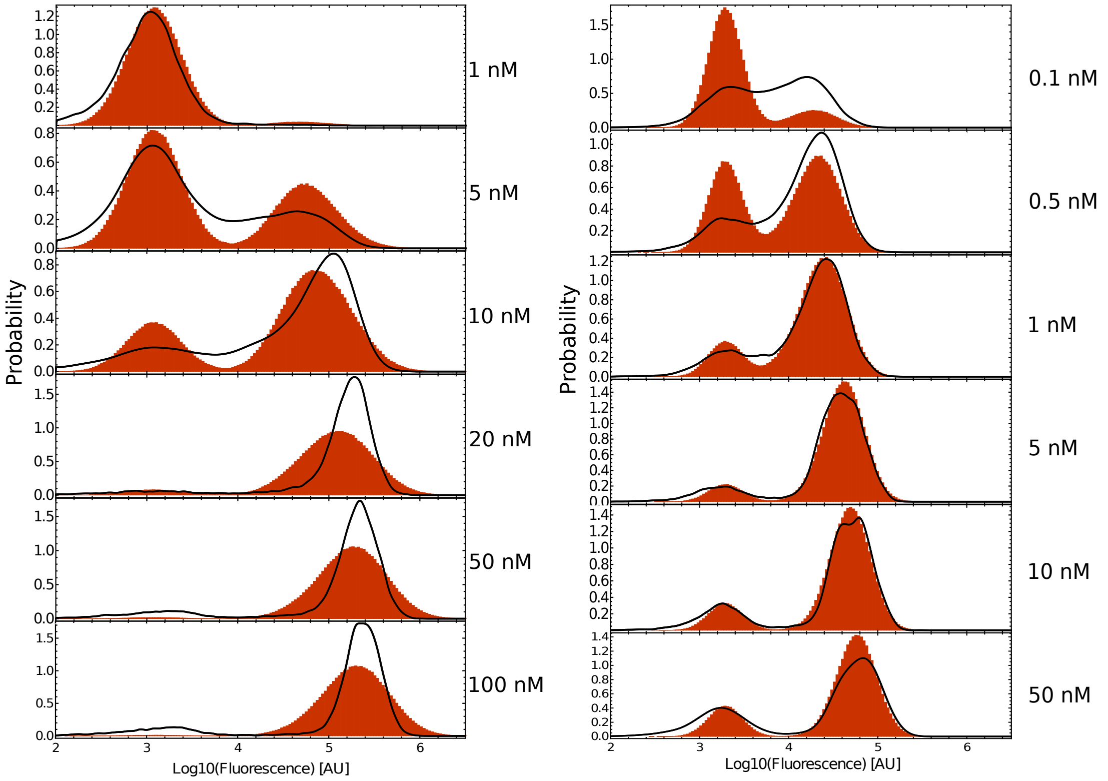

Figure S15: Comparison of the experimental results (solid line) and the stochastic simulation (histograms) for the (left) single reporter system and (right) dual reporter system. Used parameters are given in Tables S5 and S6 together with: (left): $\delta = 2.4$, $\nu = 11.6$, $\gamma = 0.001$; (right): $\delta = 0.8$, $\nu = 11.6$, $\gamma = 0.01$.

References

[1] Zalatan,J.G., Lee,M.E., Almeida,R., Gilbert,L.A., Whitehead,E.H., La Russa,M., Tsai,J.C., Weissman,J.S., Dueber,J.E., Qi,L.S. and Lim,W.A. (2015) Engineering complex synthetic transcriptional programs with CRISPR RNA scaffolds. *Cell,* **160**, 339–350.

[2] Shapiro,H.M. (August, 2003) Practical Flow Cytometry, Wiley-Liss, New York 4. edition.

[3] Thomas,P., Sekhar,A.C., Upreti,R., Mujawar,M.M. and Pasha,S.S. (2015) Optimization of single plate-serial dilution spotting (SP-SDS) with sample anchoring as an assured method for bacterial and yeast cfu enumeration and single colony isolation from diverse samples. *Biotechnology Reports,* **8**, 45 – 55.

[4] Könning,D., Rhiel,L., Empting,M., Grzeschik,J., Sellmann,C., Schröter,C., Zielonka,S., Dickgießer,S., Pirzer,T., Yanakieva,D., Becker,S. and Kolmar,H. (August, 2017) Semi-synthetic vNAR libraries screened against therapeutic antibodies primarily deliver anti-idiotypic binders. *Scientific Reports,* **7**, 9676.

[5] Schneider,C., Bronstein,L., Diemer,J., Koeppl,H. and Suess,B. (July, 2017) ROC'n'Ribo: Characterizing a Riboswitching Expression System by Modeling Single-Cell Data. *ACS Synthetic Biology,* **6**, 1211–1224.

[6] Sanft,K.R., Gillespie,D.T. and Petzold,L.R. (January, 2011) Legitimacy of the stochastic Michaelis-Menten approximation. *IET systems biology,* **5**, 58.

[7] Smadbeck,P. and Kaznessis,Y. (December, 2012) Stochastic model reduction using a modified Hill-type kinetic rate law. *The Journal of Chemical Physics,* **137**.
